# Characteristics of 351 supernumerary molar teeth In Turkish population

**DOI:** 10.4317/medoral.17605

**Published:** 2011-12-06

**Authors:** Muhammed İ. Kara, Ali M. Aktan, Sinan Ay, Cihan Bereket, İsmail Şener, Mehmet Bülbül, Şeref Ezirganlı, Hidayet B. Polat

**Affiliations:** 1 DDS, PhD Assistant Professor Department of Oral and Maxillofacial Surgery, İzmir Katip Çelebi University, Faculty of Dentistry, İzmir, Turkey; 2 DDS, PhD Assistant Professor Department of Oral Diagnosis and Radiology, Gaziantep University, Faculty of Dentistry, Gaziantep, Turkey; 3 DDS, PhD Associate Professor, Department of Oral and Maxillofacial Surgery, Gaziantep University, Faculty of Dentistry, Gaziantep, Turkey; 4 DDS, PhD. DDS, PhD Assistant Professor Department of Oral and Maxillofacial Surgery, Ondokuz Mayıs University, Faculty of Dentistry, Samsun, Turkey; 5 DDS, PhD Assistant Professor, Department of Prosthodontics, Gaziantep University, Faculty of Dentistry, Gaziantep, Turkey; 6 DDS Research Assistant, Department of Oral and Maxillofacial Surgery, Cumhuriyet University, Faculty of Dentistry, Sivas, Turkey; 7 DDS, PhD Private Dentist, Kayseri, Turkey

## Abstract

Objective: The aim of the present study was to evaluate the demographic profile of supernumerary molar (SM) teeth in people in various regions of Turkey. Study Design: A retrospective analysis was carried out on an initial sample of 104,902 subjects drawn from the ortopantographics files from 10 clinics in 7 Turkish cities with documentation of demographic data, the presence of SM teeth, their location, eruption, morphology, and position within the arch. In one region associated patho­logies and treatments were also evaluated. Results: Three hundred fifty-one SMs were detected in 288 patients, constituting 0.33% of the study subjects, with a greater frequency in females (56.4%). SMs were found more frequently in the maxilla (87.7%) than in the mandible, and distomolars (62.9%) were more common than paramolars. The SMs encountered were mostly of conical shape (45.7%), impacted (81.1%), and in a vertical position (52.1). The 33% of SM teeth were related to impacted molar teeth. Conclusion: The most common complication involving these teeth was soft tissue irritation. Demographic data from such specific extensive studies are crucial for improved diagnosis of SM teeth. Early detection allows for measures against complications and more successful therapy.

** Key words:**Supernumerary molars, distomolar, paramolar, prevalence.

## Introduction

Supernumerary teeth (ST) can be defined as any teeth or tooth substance in excess of the usual configuration of the normal number of deciduous or permanent teeth (1). The condition is also known as hyperdontia. ST may occur singly, multiply, unilaterally or bilaterally, and in one or both jaws (2,3). Although several theories have been submitted to explain their development, the precise etiology of ST is not clearly understood, but the most common view is that ST develop as a result of horizontal proliferation or hyperactivity of the dental lamina (4).

The prevalence of ST ranges between 0.45% and 3%, depending on the literature source, and is more frequent in females than in males (proportion 2:1) (5). ST may be classified topographically according to their position in the dental arch as mesiodens, distomolars (DMs), or paramolars (PMs), which are teeth situated lingually or buccally to a molar tooth (6). Although ST can be encountered in any location in the dental arch, they are commonly found on the maxillary midline, where they are called mesiodens. This location is followed in decreasing order of frequency by maxillary fourth molars (which are located distally to the third molars), maxillary lateral incisors, mandibular fourth molars, and mandibular central incisors (4,6).

ST may erupt normally, be inverted or transverse, assume an ectopic position, or follow an abnormal path of eruption. They can be diagnosed during a routine, clinical, or radiographic evaluation and sometimes are not responsible for any discernable side effects on the neighboring teeth. Nonetheless, they can cause a variety of complications including delayed eruption, noneruption, crowding, or displacement (including rotations of permanent teeth) and, less frequently, development of odontogenic cysts or resorption of neighboring teeth (7,8).

In this study we aimed to evaluate the demographic profile of supernumerary molars (SMs) in various regions of Turkey. In addition to demographic data, SM location, eruption, morphology, position within the arch, complications related to SMs, and treatment options were also evaluated. Demographic data from these studies are vital for improved diagnosis of SM as early as possible to avoid complications and ensure successful therapy. Several previous studies (8-10) have given statistics for various ST or SMs in various populations, but there was no specific extensive study considering supernumerary molar teeth.

## Material and Methods

The study was undertaken with an initial sample of 104,902 subjects drawn from the orthopantographic image files of 10 clinics in 7 different cities in Turkey. Data were collected from the northern (Samsun), southern (Gaziantep), central (Sivas, Kayseri, Tokat, and Konya), and western (Bolu) regions of Turkey. Diagnoses of SM teeth were made during clinical and radiographic examinations in Sivas but were made only during radiographic evaluations in the other regions. Thus, the Sivas region was evaluated independently. Radiographic examination of the molar region was based on panoramic radiographs independently by 5 dentists with over 5 years of experience.

For each patient we collected demographic variables including the number of SM teeth, age, and sex. Following the radiographic examination, we analyzed the characteristics of the SM including location, eruption, morphology, and position within the arch. In addition, clinical complications and treatment protocols were analyzed in the Sivas region. SMs were classified according to morphology and referred to as conical, tuberculate, or supplemental. Because odontomas are not considered SM teeth, they were excluded from the study. Regarding orientation in relation to permanent teeth, SM teeth were classified as normal (normally oriented), inverted (opposed-oriented), inclined (45-degree-oriented), or horizontal (90-degree-oriented). The ratios of the number of SMs in the maxilla and mandible and on the right and left sides were also calculated.

## Results

Age and sex

Out of 104,902 radiographs, 351 SM teeth were detected in 288 patients. The distribution of SMs according to different regions of Turkey is shown in ( Table 1). Ages of the patients ranged from 14 to 43 years, with a mean of 23.45 years. There were 153 (43.6%) males and 198 (56.4%) females, with a male to female ratio of 1:1.29. Although there was no predominance according to sex for distomolars, paramolars were more prevalent in females, with a male to female ratio 1:2 ( Table 2).

**Table 1:**
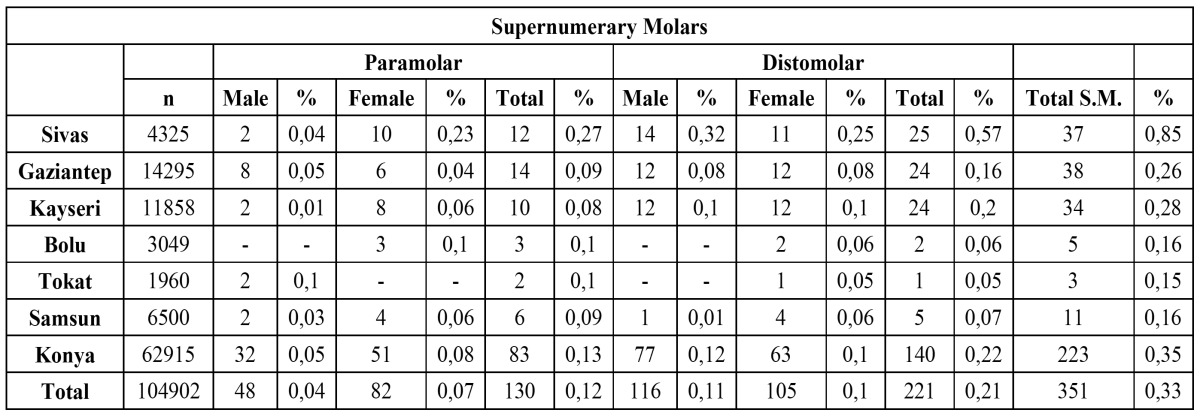
Distribution of supernumerary molars according to different location of Turkey.

**Table 2:**
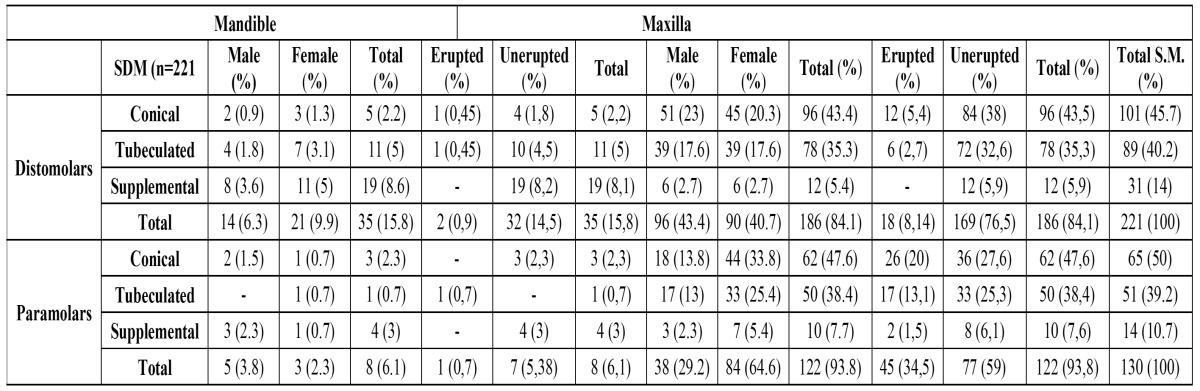
Distribution of distomolar and paramolar teeth by morphology, sex and location and their relation between eruption and morphology.

Impaction

Of the 351 SMs, 285 (81.1%) were fully impacted, and 66 (18.9%) were partially or fully erupted. In the distomolar group, 90.8% of maxillary DMs and 91.4% of mandibulary DMs were fully impacted. In the paramolar group, 63.1% of the maxillary PMs and 87.5% of the mandibulary PMs were fully impacted. Of all the PMs, 60% of the conical, 66.6% of the tuberculate, and 85.7% of the supplemental were impacted, while the corresponding rates for the DMs were 91%, 92.1%, and 100%, respectively ( Table 2).

Shape (Morphology) 

Among the 221 distomolars, the conical shape was the most frequent (45.7%), followed by the tuberculate (40.2%) and the supplemental (14%). Of all the paramolars, the majority were conical (50%), followed by tuberculated (39.2%) and then supplemental (10.7%) ( Table 2).

Location

The frequency of SMs was much greater in the maxilla than in the mandible, with a ratio of 7.16:1. Assessing distomolar location, we found that 84.2% affected the maxilla and 15.8% involved the mandible ( Table 2). In the paramolar group, supernumeries in the maxilla accounted for 93.8% of the cases. Detailed information about SM location is shown in ( Table 2).

The SMs were almost equally distributed on either side of the 2 arches.The prevalence of paramolar teeth was slightly higher on the left side (51.3%) than on the right side (48.7%), but there was no difference in the distomolar group ( Table 3).

**Table 3:**
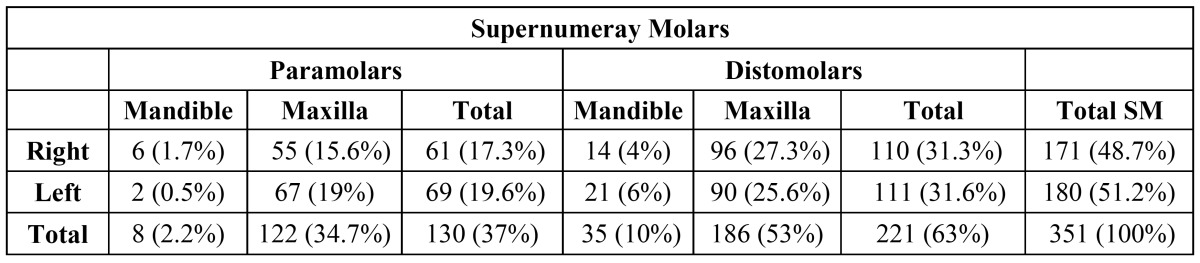
Location of supernumerary molar teeth in the jaw.

Most of the PMs (51.5%) were located around the third molar teeth (buccally, palatally, or oclusally located of third molar teeth); 32.3% were located between molars 2 and 3; 16.1% were located between molars 1 and 2.

Of the 288 patients whose SM radiographs we examined, 227 (78.8%) had a unilateral SM, 56 (19.4%) had bilateral SMs, 3 (1%) had bimaxillary SMs (each jaw having one ST), and 2 (0.7%) had multiple SMs (both of these patients had three SMs).

Orientation

Most of the SMs were normally oriented (vertical positon) (52.1%), followed by 35.9% in an inclined positon (35.9%), 9.4% in a horizontal position, and 2.5% in an inverted position. Detailed descriptions are given in ( Table 4).

**Table 4:**
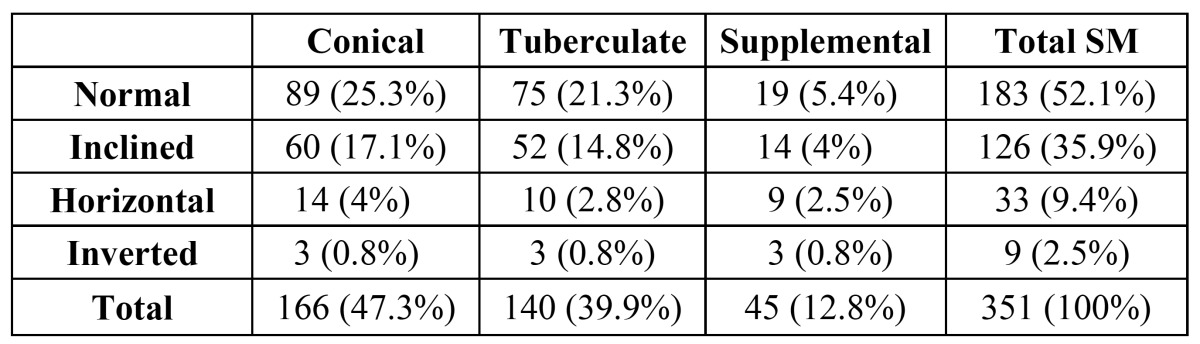
Relation between orientation and morphology of supernumerary molars.

Effect on other molar teeth

One third of the SMs (116 of 351) had affected other molar teeth, causing the related teeth to become impacted. Among the 221 distomolars, 34 teeth (15.3%) were related to impacted third molar teeth. In the paramolar group, 82 of 130 teeth (63%) were related to impacted molar teeth.

Assessment of SMs in Sivas

In the Sivas group, 30 patients with 37 SMs were referred to the Cumhuriyet University Faculty of Dentistry or a private dental hospital. Only 10 of the 37 ST (27%) had caused any complications. The main complications of SMs were soft tissue irritation in the buccal region (4 patients), pericoronitis (3 patients), follicular or cystic differentiation (2 patients), and diffuse dental caries (1 patient).

The treatment choices of these patients varied. Surgical removal of these teeth was applied to 21 of 30 patients. Because the other 9 patients were unwilling to undergo surgery, no treatment was applied, but these patients were asked to schedule routine visits to check for possible further complications.

**Figure 1:**
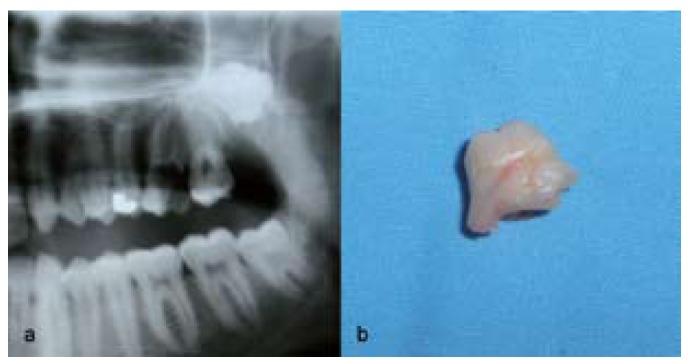
Radiographic appearance of fusion of the third molar and distomolar teeth (a), appearance of fused teeth after operation (b).

**Figure 2:**
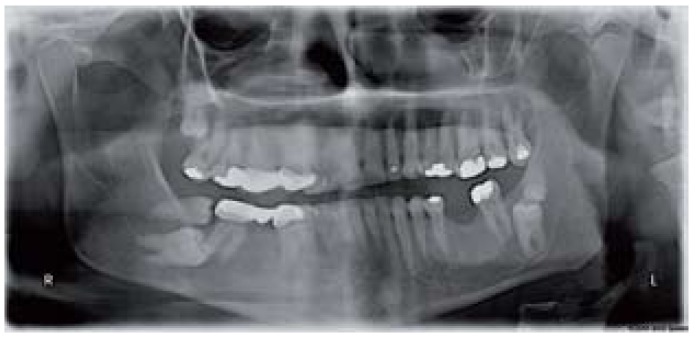
Radiographic appearance of multiple distomolar. An unusual case of kissing molars was also showed.

**Figure 3:**
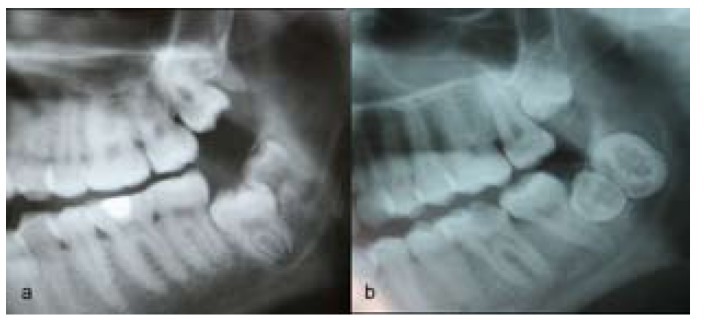
Radiographic appearance of hypomineralized distomolar tooth (a), an example of macrodontic distomolar tooth (b).

Moreover, unusual conditions were diagnosed in 5 patients in the Sivas group. These included 2 cases of fusion of third and fourth molars (Fig. 1) as well as 1 case each of kissing molars (Fig. 2), hypomineralized distomolar (Fig. 3), and macrodontic distomolar (Fig. 3).

## Discussion

Opinions about the possible cause of the formation of supernumerary teeth vary as to whether they result from continued proliferation of dental lamina or arise from the dichotomy of a tooth germ. According to the literature, most researchers accept the theory that ST originate from the budding of dental lamina or from independent dental elements (10). Because ST frequently occurs in persons with other dental anomalies and developmental disorders, it is thought that their development may be affected by a hereditary component and environmental factors (11).

ST is more frequently encountered in the permanent dentition but also can occur in deciduous dentition. Mason et al. (2000) reported that the prevalence of ST was 0.3%-0.8% in the primary dentition and 1.5%-3.5% in the permanent dentition (12). In the case of SMs, they are observed in 2% according to Luten (1967) (13). In this study the prevalence of supernumerary molars was 0.33%, which does not coincide with the findings of these studies.

Based on variations in form, three different types of ST have been described—conical, tuberculate, and supplemental teeth. The type of ST is seen as important in the literature in accordance with various possible effects on the adjacent dentition (14). Foster and Taylor (1969) reported that tuberculate types of ST more frequently caused delayed eruption, but conical types more frequently caused displacement of the adjacent dentition (15).

If supernumerary teeth resemble normal morphology, they are called supplemental teeth. These are more commonly encountered among the mandibulary distomolars, which is consistent with our data. On the other hand, tuberculate type ST are generally rudimentary in shape, are smaller in size, and display more than one cusp (10,16). Although ST is frequently normal or conical in shape in the deciduous dentition, the form of such teeth varies in the permanent dentition. Generally, the shape of supernumerary teeth appears conical in the permanent dentition (16). In our survey, the most common type of SMs were conical, which according to the literature, constitute 47.3% of all SMs. The lower frequency rates for tuberculate and supplemental SMs were 39.9% and 12.8%, respectively. Unerupted SMs were not presented with a similar frequency—all of the supplemental, 92.1% of the tuberculate, and 84.1% of the conical distomolars were impacted. On the other hand, 85.7% of supplemental, 64.7% of tuberculate, and 60% of conical paramolar teeth were impacted. Among SMs in general, 64.6% of paramolars and 90.9% of distomolars were impacted.

The inclusion of odontomas in the morphologic categories of ST is controversial. Although an odontoma may be considered a type of defective tooth development, it is frequently designated an evolutive tumor (16). Because of this uncertainty, odontomas were not included in this study.

Definitive management of patients with ST remains controversial in terms of whether to remove such teeth or to monitor them. ST can lead to delayed eruption or non-eruption, displacement of permanent teeth, occlusal disruption, cystic degeneration around them, or resorption of the roots of neighboring teeth (11). In this study, 27% of SMs in the Sivas group had caused at least one complication, including cyst formation, pericoronitis, diffuse dental caries, or soft tissue irritation. Analysis of the SM management approaches used in the Sivas group showed that surgical removal of the teeth was applied to 70% of the patients. The remaining patients were instructed to come for routine follow-up visits.

Supernumerary molar teeth were encountered at the end of the tooth series, so they were evaluated much like impacted or erupted third molars (10). In this study, 63% of paramolar teeth and 15.3% of distomolar teeth affected molar teeth. According to Fernández Montenegro et al. (2006), 14.7% of distomolar teeth affect molar teeth, which coincide with our results (17). Menardía-Pejuan et al. (2000) reported that 40% of molars are affected by SMs. In this report, 33% of all ST affected molar teeth (18).

The sex distribution reported by most authors shows males being more commonly affected in the permanent dentition (10). Mitchell and Bennett (1992) documented a 2:1 ratio in favor of males. In this study, there was no sex differentiation for distomolar teeth, but paramolar teeth were found more often in females than males at a ratio of 2:1, which does not corroborate the literature.

SMs are found more frequently in the maxilla than in the mandible. In 79% of the cases, SMs affect the maxilla, according to Grimanis` research (19). Menardía-Pejuan et al. in 2000 reported percentages of 86.8% of SMs affecting the maxilla (18). This study is consistent with the literature with 87.7% of SMs being seen in the maxilla. The orientation most of the SM teeth was vertical, followed by inclined (35.9%), horizontal (9.4%), and inverted (2.5%).

This study profiled the demographic data as well as the location, eruption, morphology, and position within the arch of 351 supernumerary teeth found in 104,902 people, making this the largest series of SMs described in the English-language literature. In addition, complications related to SMs, treatment options, and unusual cases were described for one regional group.
